# Mpox: An emerging or re-emerging infection with a potential colossal burden on healthcare globally

**DOI:** 10.4102/ajlm.v14i1.2644

**Published:** 2025-05-31

**Authors:** Chikwelu L. Obi, Nqobile M. Mkolo, Liziwe L. Mugivhisa, Modupe O. Ogunrombi, Mukhethwa M. Mphephu, Clarissa M. Naidoo

**Affiliations:** 1 Dean’s Office, School of Science and Technology, Sefako Makgatho Health Sciences University, Pretoria, South Africa; 2 Department of Biology and Environmental Sciences, School of Science and Technology, Sefako Makgatho Health Sciences University, Pretoria, South Africa; 3 Department of Clinical Pharmacology and Therapeutics, School of Medicine, Sefako Makgatho Health Sciences University, Pretoria, South Africa

**Keywords:** mpox, clades, antivirals, vaccines, laboratory diagnosis

## Abstract

The World Health Organization identified mpox (formerly known as monkeypox), as a resurgent zoonotic epidemic caused by the mpox virus. It is an emerging and re-emerging pathogen with a range of hosts and geographical distribution worldwide. Peer-reviewed scientific articles from 1958 to 29 August 2024 related to global mpox research were extracted from Web of Science^TM^ Core Collection and Google Scholar Databases to gauge the extent of the infection. Mpox is marked by a recent resurgence of infections across continents, with Africa being the hardest-hit region. The mpox re-emergence has shown a new mechanism of transmission, with several causes such as a rise in the number of unvaccinated individuals, behaviour risk factors, waning immunity, genetic evolution, and environmental circumstances. Preventive and control measures of mpox include vaccination and patient isolation, while treatment involves antivirals and antibiotics for secondary bacterial infections. Laboratory diagnosis entailing polymerase chain reaction can be effective for routine purposes, but results of serological tests must be interpreted with caution, because of cross-reacting determinants among orthopoxviruses. The structure and classification of the mpox virus, clinical manifestations, pathophysiology, epidemiology, historical antecedent, therapeutics, vaccines, and laboratory diagnosis of the disease are explicated, showcasing mpox as an emerging or re-emerging infection with a potential colossal burden on healthcare, and its classification as an international public health emergency by the World Health Organization.

**What this study adds:**

This review provides the global situation of mpox as an emerging or re-emerging infection, warranting its designation as an international public health emergency.

## Introduction

Mpox, formerly known as monkeypox by the World Health Organization (WHO), is a zoonotic viral infection with a cycle of transmission involving the active participation of wild animals.^1^ The mpox virus is regarded as both an emerging and a re-emerging pathogen with a varied range of hosts.^2^ The re-emergence of the mpox virus version with enhanced virulence since its first identification and reporting in monkeys in 1958 is favoured by the alterations in the virus genome resulting from mutations.^2^ Although mpox was identified 70 years ago, it has been ignored in medical literature because of its rarity.^3^ However, it has recently been accorded much attention because of a trail of morbidity and mortality in different age groups worldwide, although its foci were mainly in Africa.^2,4^

The mpox name is likely to have originated from the fact that mpox was initially identified in monkeys (cynomolgus) from Singapore used for research purposes in 1958 and then transported to Copenhagen in Denmark.^3,5,6^ Mpox is caused by a diverse and large group of orthopoxviruses^7^ and is characterised by symptoms that resonate with those of smallpox and chickenpox, even though mpox usually presents milder symptoms compared to smallpox and has a 10% fatality rate in people who are not vaccinated against smallpox.^3^ Mpox is a viral disease that has been added to the WHO list of communicable diseases by viruses with the possibility for widespread and epidemic repercussions and fears after the coronavirus disease 2019 outbreak, hence its classification as an international public health emergency.^8,9,10^ By 29 August 2024, a total of 89 596 established cases of mpox had been recorded across 114 countries, with 157 deaths,^11^ resulting in apprehension regarding the geographic spread.^12^

Even though the exact causes and reasons for the re-emergence of mpox are unknown, it is postulated that it could be a result of several factors, such as the increase in the number of people not vaccinated against smallpox, risk factors of behaviours of men having sex with other men, decreasing immunity, genetic evolution, and ecological conditions.^11^ The global spread of the disease is a health hazard.^13^ Additionally, the recent disease modelling proposes that pandemics which are usually caused by emerging diseases are expected to surge in severity and frequency in the upcoming decades.^14^ Hence, the review aims to report on mpox as an emerging or re-emerging infection with a potential colossal burden on healthcare.

## Methods

On 29 August 2024, peer-reviewed scientific articles related to global mpox research were extracted from the Web of Science^TM^ Core Collection and Google Scholar Databases. The search included papers from 1958 to 29 August 2024, and included phrases associated with mpox, monkeypox virus, mpox classification and structure, mpox epidemiology, mpox vaccines, and mpox laboratory diagnosis. In every study that was retrieved, only English-language literature was included.

## Classification and structure of the mpox virus

### Classification of mpox virus

The mpox virus belongs to the *Poxviridae* family, which comprises 22 genera and 83 species.^15^ The family is subdivided into two subfamilies, namely *Chordopoxvirinae*, with 52 species and 18 genera, and *Entomopoxvarinae*, with 31 species and 4 genera. Twelve members of the genus Orthopoxvirus have been recognised to affect both humans and animals.^15,16^ The variola virus is the commonly known member that causes smallpox, while others are known as the Cowpox virus, Camelpox virus, Skunkpox virus, Volepox virus, Taterapox virus, Akhmeta virus, Abatino macacapox virus, Raccoonpox virus, Vaccinia virus (VACV), Ectromelia virus, and Mpox virus.^15,16^

### Structure of mpox virus and its genome

**FIGURE 1:**
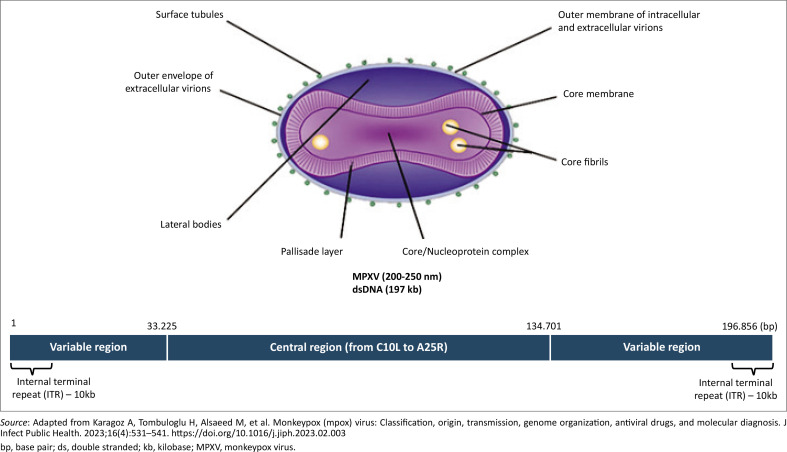
The structure of the mpox virus and its genome.

The lipoprotein outer membrane of the mpox enveloped virus is oval or brick-shaped.^17^ The normal size of the mpox virus varies from 200 nm to 250 nm, with a double-stranded DNA genome of almost 197.2 kb, and encodes 181 proteins.^18^ The hairpin end of the linear genome is covalently closed and there are no free 3’ or 5’ ends.^18^ The genome ends consist of the 10 kb inverted terminal repeats and nucleotide homopolymers (Figure 1). However, short tandem repeats have been observed in the genome.^18^ Orthologous poxvirus genes are closely packed, and intergenic areas that are 100 bp in length are uncommon.^18,19^ The ‘housekeeping’ proteins that are encoded by orthologous poxvirus genes in the conserved central area are responsible for transcription, replication, and virion processes. In the terminal domains, the proteins encoded by orthologous poxvirus genes are linked to the pathogenesis and host range.^19,20^

**FIGURE 2:**
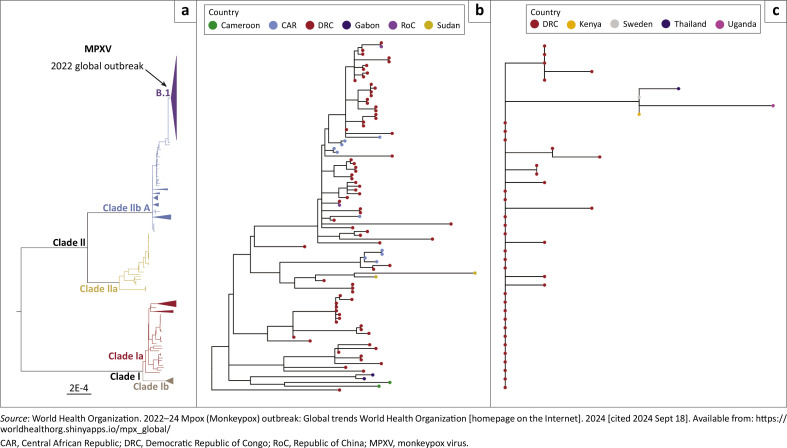
Phylogenetic visualisations of mpox virus clades generated with *ggtree* package: (a) all MPXV clades, (b) Clade Ia and (c) Clade IIb.

The mpox virus consists of two main clades: Clade I, formally identified as the Congo Basin clade, and Clade II, formally recognised as the West Africa clade.^21,22^ These clades are split into two subclades, namely Clade Ia and Clade Ib in Clade I, and Clade IIa and Clade IIb in Clade II (Figure 2).^21,22^ Lately, Clade III, consisting of hMpox-1 A, B.1, A.1.1, A.1, and A.2, has been reported in non-African countries, including Mexico.^22,23,24^ Clade III varies primarily in regions of coding and is linked to the host’s recognition of antigenic determining factors and immune modulation.^25^ A total of 46 single-nucleotide polymorphisms are reported from Clade III.^23,26^ The mutation of mpox is because of the action of the apolipoprotein B mRNA editing enzyme, catalytic polypeptide 3 family of cytosine deaminases.^27^

## Pathogenesis and pathophysiology of mpox virus

**FIGURE 3:**
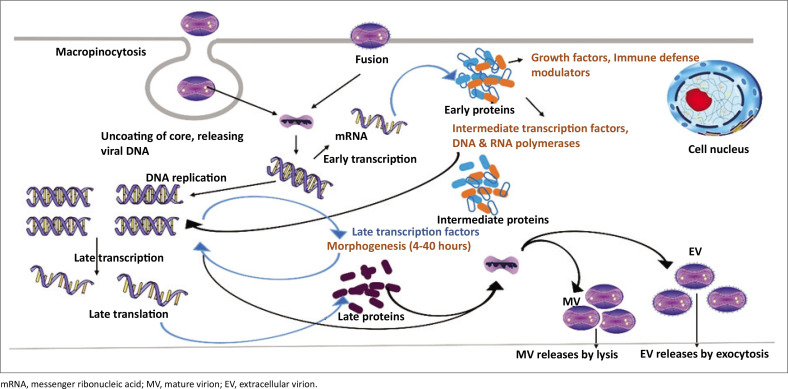
Cytosolic mpox virus pathways depicting the enveloped virion entry to the host cell through fusion and the mature virion.

Viral endocytosis, cell membrane fusion, and micropinocytosis accelerate viral transmission through oropharyngeal, subcutaneous, nasopharyngeal intramuscular, and intradermal pathways (Figure 3).^28^ The virus replicates at the location of inoculation, which leads to the virus spreading to different organs of the lymph nodes, blood, bone marrow, tonsils, and spleen, triggering inflammatory immune-mediated phagocytosis.^29,30^ This signifies the period of incubation, which usually lasts for 7 days to 21 days. Through the guidance of the enveloped and mature virions, the genome and proteins of the mpox virus are discharged into host cells. Intracellular mature virions encompassing the DNA encoding the virus get generated when the virus mRNA transcription and translation occurs.^31,32^ Intracellular mature virions enfolded in the Golgi apparatus fuse with the host inner cell membrane to form cell-related virions before being released into extracellular areas to produce extracellular enveloped virions.^29^

The mpox virus can be spread in different modes from animals to humans, or humans to humans. However, the general mode is through human contact with the bodily fluids of the infected animals.^33,34,35^ The distinction between mpox and smallpox is that mpox virus infection triggers lymphadenopathy in humans while smallpox does not. The early symptoms of mpox virus infection are muscle aches, headache, diarrhoea, fever, chills, vomiting, fatigue, and backache, which advance to fatigue, developing into secondary bacterial infections of the skin or blood and lung infections.^21^ The body of the infected person can be affected by lesions that are initiated in the oropharynx, and inflammation of the heart, brain or other organs can also occur.^36,37^ Moreover, patients can also have neurological concerns in the form of encephalitis.^36^ The onset symptoms occur mostly in all age groups, although infants or the elderly might have a less frequent or different presentation of symptoms.^21^

## Epidemiology and historical outbreaks

Mpox virus was identified first in monkeys in 1958 during the occurrence of outbreaks in colonies of captive monkeys that were kept for purposes of research in a Danish research institute, and has since evolved into a zoonotic infection affecting the human population.^37,38,39,40^ The first detection of mpox in a human host was discovered on 01 September 1970 in a baby (9 months old) in the Democratic Republic of the Congo.^38,39,40,41,42^ Since the campaign for worldwide smallpox vaccination gradually ended between the 1970s and 1980s, and smallpox was eventually eradicated by 1977, the number of human mpox cases in Africa has been on the increase.^43,44,45,46^

The WHO verified a case of mpox later in 1980, and since then the virus has spread globally with a ten-fold increase in the past decades.^38,39^ In 1982, active mpox surveillance programmes were started in the Democratic Republic of the Congo. This increased the number of detected cases. A high number of approximately 386 cases was observed in the Democratic Republic of the Congo alone, whereas only 18 cases were observed in other endemic countries, with children being most affected.^47,48^

In 1986, mpox surveillance programmes in the Democratic Republic of the Congo were terminated because of the onset of another epidemic, termed AIDS, in Africa. This prompted the WHO to redirect its resources on public health.^49,50^ There was a decline in the number of confirmed human mpox cases in the decade that followed. No case was reported to the WHO beyond 1992. It was only in 1996 and 1997 that a cluster of 344 cases were reported in the Democratic Republic of the Congo among a cohort predominantly unvaccinated against smallpox.^51^ Since then, outbreaks have been common in the Democratic Republic of the Congo. Annually, over 1000 cases have been reported since 2005.^44^

In Nigeria, there was a complete hiatus of cases for 39 years until 2017, when mpox re-emerged. A sizable country-wide outbreak of over 120 laboratory-confirmed or -suspected infections involving Clade II was then recorded.^51^ Several factors, including population density, shifting patterns of land use, and declining herd immunity provided by smallpox vaccination were reported to be providing opportunities for more zoonotic transmission and outbreaks.^51^ In contrast, between 1970 and 2018, case reports of human mpox infection from other countries were infrequent.^43,44^

Mpox re-emerged as a global outbreak in May 2022, with almost 86 000 cases and 53 deaths confirmed from 110 countries around the world, especially in areas where the disease had not been common.^13^ In 2023, there was an occurrence of mpox in the United States^3^ with 47 human cases that might be a result of close interaction with prairie dogs which were alleged to have been infected during a shipment imported from Ghana.^1^ A recent outbreak was also reported in Europe and Africa, with suspected cases and total deaths recorded across different countries between 2022 and 2024. The African region experienced a surge in cases in July 2024. These data outline the outbreak trends across various geographical locations from 2022 to 2024.^21,22,23,24,25,26,27,28,29,30,31,32,33,34,35,36,37,38,39,40,41,42^

The geographical location and demographics revealed mpox occurrences mainly in Central, East, and West Africa, as well as multi-country outbreaks for Clade IIb, lineage B.1. Demographics indicated morbidities of 70% and above, except for adults in Burundi, across all clades and sub-clades, with a preponderance in men (Online Supplementary Table 1). Sexual intercourse mode of transmission is reported to contribute more to the spread of the virus, especially by men who have sexual intercourse with other men.^21^

**FIGURE 4:**
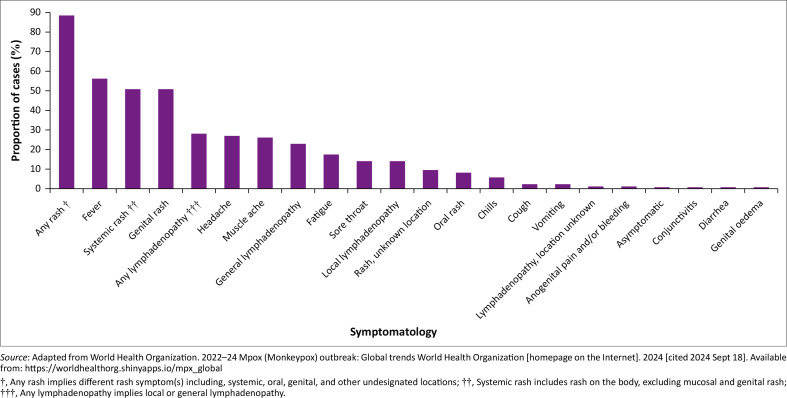
Symptomatology of people infected with mpox, reported from January 2022.

Previously, the age of people infected with mpox ranged from 7 months to 40 years,^47^ where more than 80% of the mpox cases were seen in children under 10 years of age.^47^ The attributable deaths (17%) also occurred in children.^47^ The median age of the affected people moved from younger children of 4 years old in the 1970s to the median age of young adults of 21 years between 2010 and 2019.^3^ This could be because of the suspension of smallpox vaccinations which offered some form of herd immunity against mpox.^3^ However, recently in 2024, the age of people infected with mpox ranged from 0 years to 65 years, and men between 18 years and 44 years old continued to be excessively affected by the outbreak, accounting for 79.2% of reported cases, while women account for 3.6%. Moreover, a rash (systemic, oral, genital, or unknown location) is reported in 88% of cases with at least one reported symptom, and is thus the most common symptom (Figure 4).^21^ It was reported that the denotation of a symptom may differ throughout reporting systems in different countries as a result of a general lack of negative reporting and symptom classifications.^21^

## Management, potential therapeutics, and vaccines

The treatment of mpox infections involves community-based and clinical measures to limit its spread.^52,53^ In community and clinical settings, patients should stay at home, and avoid contact with infected people, and healthcare professionals should follow guidelines to reduce the risk of infection.^8,54^ The guidelines include avoiding direct contact with skin lacerations, treating skin rashes with personal protective equipment, and wearing gloves.^55^ Infected individuals should be isolated for approximately 2–4 weeks to decrease the spread of the virus and mitigate the impact of possible outbreaks.^56^ In addition, supportive care is essential for patients with limited antiviral therapies, including hydration, nutrition, symptomatic management, and antibiotic treatment.^57,58,59^ In immunocompromised patients, superinfection and eye infections can be managed with antibiotics.^60^ Intensive care management and mechanical ventilation are often used for respiratory and neurological issues.^61^

### Potential antiviral agents

New antiviral agents and vaccines such as cidofovir (Gilead Sciences, Foster City, California, United States), brincidofovir (Chimerix, Durham, North Carolina, United States), tecovirimat (SIGA Technologies, New York, United States), immunoglobulin (Vaccinia Immune Globulin; SIGA Technologies, New York, United States) ACAM2000® (Emergent BioSolutions, Gaithersburg, Maryland, United States), and JYNNEOS^TM^ (Bavarian Nordic, Kvistgård, Denmark) offer new therapeutic prospects for orthopoxviruses (Online Supplementary Table 2).^62,63^ Smallpox (variola virus), mpox, and cowpox share collective genetic similarities, which include shared surface proteins such as antibodies, and T-cell receptors.^62^ In addition, some smallpox vaccines create antibodies against a range of epitopes that bind to the shared epitopes of mpox proteins.^63^

The antiviral resistance of cidofovir is slow, affecting poxviruses through serial passage.^63,64,65^ It is effective in treating compound orthopoxvirus infections and is used in humans for poxvirus infections, molluscum contagiosum, and AIDS-associated cytomegalovirus retinitis.^66,67^ Brincidofovir, a lipid-conjugated cidofovir analogue, was United States Food and Drug Administration (FDA)-approved in 2021 for smallpox therapy because of its comprehensive activity against double-stranded DNA viruses and lower EC_50_ compared to cidofovir.^68,69^ Its lipophilicity allows efficient entry into host cells and a prolonged intracellular half-life.^69,70^ Oral brincidofovir has shown potential in treating poxvirus infections and pre-proactive therapy of adenovirus viraemia.^71,72,73,74,75^ Tecovirimat, FDA-approved in 2018, targets the *V061* gene in cowpox and the membrane protein p37, which is responsible for extracellular enveloped virus formation.^76,77^ It is available in intravenous and oral forms and has no documented cross-resistance.^78,79^ It is effective in treating orthopoxviruses in animal models, preventing death, and reducing viral distribution to distant tissues.^80^ Tecovirimat is used to treat orthopoxvirus infections in human patients because of its tolerance levels. Four clinical trials are ongoing to evaluate its safety, tolerability, and pharmacokinetics.^81^ Vaccinia Immune Globulin Intravenous (Emergent BioSolutions, Gaithersburg, Maryland, United States) is an FDA-approved immunoglobulin used to manage smallpox vaccination and adverse effects in skin disorders.^82^ It provides passive immunity but is not recommended for immunocompromised patients, diabetic renal complications, or sepsis history.^83^ Clinicians may consider Vaccinia Immune Globulin Intravenous for complex mpox infections or T-cell immunodeficiency patients; however, live attenuated vaccines should be avoided 3 months after vaccination.^84^

### Vaccines and immunisations

First-generation smallpox vaccines were effective, but adverse effects led to subsequent vaccine development.^85^ Currently, three licensed mpox vaccines are available: modified vaccinia Ankara-BN (Bavarian Nordic, Kvistgård, Denmark), LC16-KMB (Konica Minolta, Tokyo, Japan), and OrthopoxVac (University of Florida, Gainesville, Florida, United States).^86^ The FDA has authorised ACAM2000 and JYNNEOS for smallpox prevention and cross-protective immunity.^87^ The VACV can be treated with vaccinations, either before or after exposure.^88^ Post-exposure prophylaxis should be administered 4 days or less after exposure, to prevent symptomatic infection.^89^ If mpox symptoms are absent, post-exposure prophylaxis can be administered up to 14 days after exposure.

The FDA authorised JYNNEOS intradermal injection as a route of administration for emergency use in August 2022, in addition to the previously approved subcutaneous injection.^90,91^ The JYNNEOS vaccine, approved by the FDA for alternative use in mpox virus-infected persons, works by eliciting humoral and cellular immune responses.^92^ Preclinical research indicates that mpox vaccination is safe during pregnancy and breastfeeding, although there is no preclinical research available for paediatric patients, despite being safe in immunocompromised people such as HIV patients.^93^

The ACAM2000 is a licensed vaccine for smallpox and mumps, given in single doses and lyophilised for long-term storage.^93,94^ It aims to balance the risk of pathogenic mpox with potential side effects from replicative vaccinations. Furthermore, ACAM2000 is not recommended for HIV-positive patients since they have immunosuppressed systems, as it is a live vaccine that can cause severe complications. Attenuated VACV, also known as modified vaccinia Ankara, requires two injection treatments. Live VACV immunisation has been linked to skin infections, premature delivery, congenital abnormalities, stillbirth, and perinatal mortality.^94^ However, VACV immunisation has a safer profile and fewer side effects compared to ACAM2000.^95,96^ Healthcare professionals and laboratory people who are at a high risk of exposure are also evaluated for pre-exposure prophylaxis.^84^

### World Health Organization guidelines on therapeutic interventions and vaccines for mpox

Pre-exposure immunisation for high-risk persons and post-exposure vaccination within 4–14 days is suggested by the WHO’s normative recommendations for mpox, to avoid or minimise the seriousness of the disease.^21,90^ Vaccination should be used in combination with public health interventions such as contact tracing, isolation, and surveillance.^10,21^ Target Product Profiles, which outline required vaccination qualities, have also been produced by the WHO.^21,94,95^ Clinical administration places significance on supportive care related to medicine, and antivirals according to WHO guidelines.^21,54,62,63^ To ensure the reasonable allocation of vaccinations, treatments, and diagnostics, the WHO has also set up access and distribution processes, and has modified its recommendations in response to new information.^21,50^

## Laboratory diagnosis of mpox

A critical factor in the diagnosis of infections, including the mpox virus, rests on laboratory diagnosis.^96,97,98,99^ Two of the definitive methods for the laboratory diagnosis of mpox infection are the direct and indirect methods. For the indirect method, specimens collected are screened for the virus, the nucleic acid, or the viral antigens. To identify the DNA, nucleic acid amplification testing is employed for the direct method. On the other hand, immune responses to the viral antigens form the basis for the detection using the indirect method.^98^

### Specimen collection, transport, and storage

The main sample for laboratory diagnosis of mpox infection is the skin lesions, such as swabs from lesion exudate or lesion crusts. Ideally, lesions, crusts, and vesicular fluids should not be placed in a similar tube, to obtain good DNA or prevent inhibitors.^97,98,99^ Oropharyngeal swabs, rectal or genital swabs, semen, or urine may be collected based on clinical manifestations.^98,99^ Blood samples are also used, especially when treated with ethylenediaminetetraacetic acid. This process may increase the concentration of the virus; however, it may not align with samples collected from lesions, as viraemia typically occurs during the early stages of infection or the prodromal phase. Sample collection should be done by trained healthcare personnel in an appropriate clinical or field setting, using personal protective equipment, and then transported to laboratories with the appropriate level of biosafety for analysis.^35,100^

After collection of samples for laboratory diagnosis of mpox, samples should be placed in the fridge at 2 °C – 8 °C or kept chilled at –2 °C within an hour after collection, before transportation to the base laboratory. Emphasis is usually placed on the storage and handling of specimens while being transported. If it is anticipated that it may exceed 7 days before processing of specimens following transportation, such samples must be stored at –20 °C or a lower temperature (± 2 °C), or at –70 °C if storage will exceed 60 days after collection, to obviate false negative results caused by, for example, inability to extract DNA.^98,99^

### Virus isolation

The first isolate of the mpox virus was obtained in 1958 from cynomolgus monkeys using monkey kidney (Vero) and human amnion cells.^101^ In humans, the virus was first isolated from a patient with a skin infection resembling smallpox. This was achieved by infecting pig embryo kidney cells, *Homo sapiens* epithelial carcinoma cells, and African Green Monkey kidney cells, where cytopathic effects were observed.^102^

Although research laboratories have employed culture-based techniques for the identification of the mpox virus, the routine identification of the virus is not recommended by the WHO as it is not only laborious, but has low sensitivity and takes some days, and must be performed using a biosafety level-3 safety chamber.^97,98^

### Electron microscopy

The use of electron microscopy is a known method for identifying structures, including the fine details of the virus after isolation. This method reveals that the mpox virus has a brick-shaped (200 nm – 250 nm) or ovoid format, displaying intricate internal structures such as a double-stranded DNA genome (∼197 kilobases) and enzymes. Although electron microscopy is useful in revealing progeny virions in different phases of assembly, immature and mature ones, in infected cells, it is not recommended for routine laboratory diagnosis of the mpox virus.^102,103,104^

### Serological methods

The use of serology to ascertain immune responses, immunoglobulin M and immunoglobulin G antibodies to mpox infection by use of enzyme-linked immunosorbent assay, plaque reduction neutralisation test, complement fixation test, haemagglutination inhibition, and immunofluorescence are all of diagnostic value. However, results obtained should be interpreted with caution because of cross reactions or shared antigenic determinants among the orthopoxviruses or, in recent cases of vaccination, against smallpox.^105,106^

### Clinical laboratory findings and biomarkers

To complement laboratory methods, variations of biochemical and haematological indices, such as thrombocytopenia, rated as the most common, hypoalbuminaemia, leucocytosis, and increased transaminase level are critical.^1^ Other findings indicate that higher levels of aspartate aminotransferase, and alanine aminotransferase may be related to poor prognosis. In addition, cytokine modulation has been reported to correlate with the severity of mpox in humans.^107,108^ The cytokines comprise interleukins 1B, 2R, 4-8, and 13, among others.^98,108^

The polymerase chain reaction (PCR) test on lesions was, however, reported to give the highest clinical sensitivity, 91% – 100%,^109,110,111,112^ whereas the sensitivity of saliva, nasopharyngeal swab, and oral swabs was between 68% and 100%, seminal fluid was 78% – 100%,^109,111^ and rectal swabs were 78% – 97%.^112^

### Genome sequencing

For epidemiological purposes or monitoring of transmission patterns as an adjunct to conventional laboratory analysis of mpox infection, whole genome DNA sequencing is critical for observing differences in the viral genome over time. Genome sequencing is not recommended for routine diagnosis due to its expensive outlay, high cost of reagents, and the advanced training required for the process. Few samples of patients may require genome sequencing from samples as genome monitoring of circulating types may assist policymakers and healthcare personnel in terms of decision-making as well as the introduction of measures to reduce or abrogate the chain of transmission.^99,113,114^

### Real-time polymerase chain reaction

It has been reported that real-time PCR is the gold standard molecular method for lab-based diagnosis of mpox.^35,97,98^ The real-time PCR workflow for mpox detection involves several key steps: denaturation, annealing, extension, and fluorescence readout. Initially, the double-stranded DNA is denatured to separate the strands into single strands, which facilitates the binding of primers to their respective target regions. The forward and reverse primers are designed to bind to specific sites on the single-stranded DNA. The reverse primer plays a critical role in this process by binding to the complementary strand, enabling the synthesis of the complementary strand during the extension phase. During annealing, both the forward and reverse primers bind to their target sequences on the DNA. Following this, DNA polymerase synthesises the complementary strand in the extension phase. The fluorescence signal is then released, allowing for real-time detection of the target sequence. Regarding other tests involved, this real-time PCR assay specifically measures the amplification of target DNA sequences, such as those within the *G2R, B7R, B6R, N3R,* and *TNF* receptor genes in the mpox virus genome, as previously reported.^113,115^ Additionally, multiplex real-time PCR assays can be used to detect and differentiate various infectious agents and different subclades (IIa, IIb) of the mpox virus.^113,114,115,116,117,118,119^

### Loop-mediated isothermal amplification

Real-time PCR is a standard technique method for diagnosing mpox infection; however, its shortcomings comprise sample processing time, constant availability of electricity supply, and technical skills.^113,120^ Consequently, in terms of point of care, a user-friendly, simple, inexpensive, and rapid method for the laboratory diagnosis of mpox in low- to middle-income countries or areas with simple laboratory infrastructure are warranted. Loop-mediated isothermal amplification (LAMP) is a recommended technique for the amplification of nucleic acid at a single and isothermal temperature of about 60 °C – 65 °C. While LAMP requires less equipment than real-time PCR, it still relies on electricity, which may limit its utility in areas with inconsistent power supply. Nevertheless, its simplicity and low-cost nature make it a valuable option for resource-limited settings.^121,122,123,124,125,126,127,128^

### Recombinase-based isothermal amplification assays

Recombinase-based isothermal amplification assays are also isothermal amplification methods involving enzyme-based DNA amplification at a constant temperature of around 37 °C to 42 °C within 10 min,^127^ and, because of its simplicity, high sensitivity, and rapidity, this method is suitable for point-of-care or field applicability for mpox detection.

### Sensors

Sensors are also used in laboratory diagnosis based on their ability to respond to stimuli, biological, physical, and chemical agents that elicit observable effects that are measurable through electrochemistry, colourimetry, and fluorescence. Their applications are simple, rapid, inexpensive, and adaptable for use in rural or less-resourced settings.^129^ In terms of the method, the RNA sensors or toehold switches can stimulate the translation of a reporter gene, such as beta-galactosidase or a green fluorescent protein, against a receptor RNA trigger sequence present in the sample. This results in the activation of a reporter protein, followed by a light reflection that generates an observable colour change. Alternatively, fluorescence-based methods may use fluorescent dyes such as 6-Carboxyfluorescein or SYBR Green to detect the presence of the target RNA, generating a measurable fluorescence signal.^129,130,131^

## Prevention and control

For effective control of mpox outbreaks, rapid recognition and investigation of new cases, along with a comprehensive understanding of all possible routes and modes of transmission, are essential strategies for vaccine distribution.^7^ Prevention of transmission of the mpox virus principally depends on hygienic practices and restrictions on contact with wild animals as the primary transmission mode of the virus to humans.^1,84^ Direct contact with an infected animal should be prevented to avoid the animal-to-human route. Additionally, physical contact with other people who are infected should be avoided, personal protective equipment should be used to reduce transmission, and there should be isolation of patients with mild cases of the disease.^9^ In cases where a person presents with lesions caused by mpox around the genitals, sexual contact must be avoided, or condoms should be used to avoid transmission.^13^

Although the majority of mpox cases during the 2022 outbreak occurred in men who have sex with men, the infection can affect individuals of all genders and age groups. During pregnancy, special precautions are necessary to prevent vertical transmission from mother to child through the placenta. Additionally, perinatal transmission can be avoided by preventing neonatal contact with mpox-related genital lesions during childbirth.^131,132,133,134^

Other avenues of transmission include tourism and travelling.^132^ Hence, prevention and control of the mpox virus can also be affected by controlling international air travel volume and entrenching policies regarding entry into countries. Deng et al.^40^ showed that a reduction in the volume of air travel and strict policies on border entry contributed to a reduction of mpox introductions of infected persons into China.^13^

Smallpox vaccine has been shown to provide almost 85% protection against mpox because of herd immunity.^11^ Pre- and post-prophylaxis vaccination has also been recommended by the healthcare authorities for healthcare workers, laboratory workers and technicians, response teams against outbreaks, as well as scientists who research clinical samples, and vaccinators.^84^ Post-exposure prophylaxis is suggested when there has been unprotected contact with infected people or there has been sharing of close spaces with infected people for extended periods (prolonged or repeated close contact) where there might be secretions of aerosols and the presence of the virus in the air particles.^84^ Vaccines, antiviral agents, and drugs such as tecovirimat are used as the first line of treatment.

## Challenges and future perspectives

Mpox prevention, treatment, and control face several challenges, including healthcare gaps and the limited availability of treatments, vaccinations, and resource environments.^135,136^ Diagnostic challenges, such as access to advanced technologies, can hinder timely and accurate diagnosis.^137^ Additionally, fever-like illnesses accompanied by a rash may be misdiagnosed as mpox.^49^ To effectively control mpox, strong public health systems are required to monitor and manage outbreaks, and trace contacts. However, underdeveloped systems in endemic areas make containment difficult.^138^ Public mistrust of vaccines, especially in poorly literate areas, can impede mass vaccination campaigns. Immunisation resistance, motivated by misleading information, remains a significant barrier.^139^

## Recommendations

The following recommendations are of significance in the narratives concerning mpox:

- Global emergency preparedness to tackle the potential pandemic of the disease is critical and should be actioned.
- Training and re-training of healthcare professionals on the differential diagnosis and other intricate aspects of the disease.
- Enhanced research activities to uncover emerging clades and their genomes, more insights into their epidemiology, and genomic surveillance to identify changes and adjust vaccines should be prioritised.
- Increased provision of vaccines, especially to endemic areas to curb centrifugal spread, should be central to the strategies to tackle the disease.
- Increased allocation of resources by countries in a bid to contain the menace of the disease and its anticipated health burden.
- Development of simple screening and diagnostic test kits or methods especially for use in less-resourced countries.

## Conclusion

Mpox is not a new disease, but an emerging and re-emerging disease caused, probably, by increased rates of zoonoses, deforestation, food insecurity, global mobility, and increased socialisation pressures. Others include risky behaviours and decreased immunity to the smallpox virus, which shares cross-reactive antigenic determinants with the mpox virus, resulting in waning protection offered by herd immunity against mpox. The key drivers of infections across various age groups and countries over the past years were the various clades. The historical foci of the disease have been mainly in Africa, in addition to reported cases in other continents, indicating varying degrees of morbidity and mortality in all affected countries. Management of the disease may depend on the range of clinical manifestations, including the use of antivirals, and antibiotics for secondary bacterial infections. Preventive and control measures are predicated on the isolation of patients, protective sexual intercourse, avoiding contact with fluids of infected persons, and vaccination, among others. Laboratory diagnosis involves the use of PCR for routine purposes, but the use of serological tests should be interpreted with caution because of shared antigenicity among the orthopoxvirues. Electron microscopy and genome sequencing are mainly for research-related endeavours.
